# Complete Laparoscopic Nephroureterectomy for the Upper Urinary Tract Urothelial Carcinoma in a Female Patient with Severe Senile Kyphosis: An Initial Case Report

**DOI:** 10.1089/cren.2015.0018

**Published:** 2015-11-01

**Authors:** Hidenori Zakoji, Tatsuya Miyamoto, Satoru Kira, Norifumi Sawada, Yuko Ootake, Hiroshi Shimura, Takahiko Mitsui, Masayuki Takeda

**Affiliations:** Department of Urology, Faculty of Medicine, University of Yamanashi, Chuo, Yamanashi, Japan.

## Abstract

Kyphosis is usually described as the deformity of the spine that results in an abnormally round back. Patients with kyphosis form a challenging group to laparoscopic surgeons because of difficulties in positioning and abdominal approach. The narrow abdomen causes difficulty with trocar insertion and some operative procedures. Here we report the transperitoneal complete laparoscopic nephroureterectomy in a case of kyphosis. An 81-year-old woman underwent this operation for urothelial carcinoma of the upper urinary tract. We took best care of the positioning and the trocar insertion; we could have accomplished utilizing four ports at semilateral position without any troubles and complications. This procedure is safe and feasible, and not thought to be a contraindication for the patients with senile kyphosis.

## Case History, Physical Examination, and Diagnosis

**FIG. 1.**
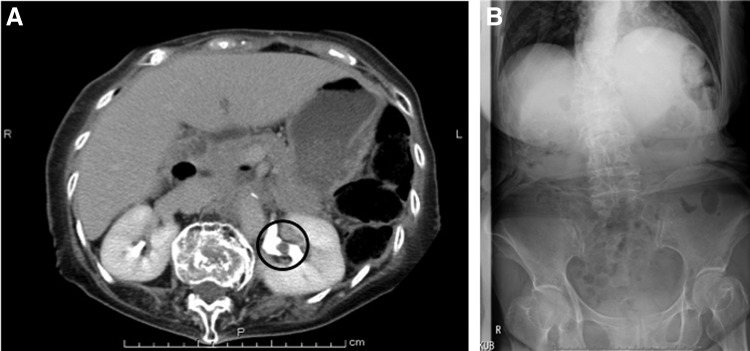
**(A)** CT scans showed a slightly enhanced mass in the left renal pelvis. **(B)** Radiography showed curvature of the spine and well-creased abdomen. Urothelial carcinoma (indexed in the *black circle*) was suspected.

The patient was an 81-year-old woman who was presented to the hospital because of macrohematuria. She was found to have the upper urinary tract urothelial carcinoma by contrast computed tomography (CT) and positive urinary cytology. The CT scan showed enhanced irregular mass in the left renal pelvis (Fig. 1A) with no distant metastasis. Cystoscopy revealed no tumors in her bladder. The patient with a height of 132 cm and a weight of 43 kg had severe senile kyphosis in succession to compressed fracture of lumbus due to osteoporosis (Fig. 1B) and could not be placed in supine position. The anteriorly tilting thoracic cage results in low-lying costal margin and a small abdominal space, however, no restrictive or obstructive pulmonary disease was found. The patient was counseled regarding the available treatment options and an informed consent for laparoscopic nephroureterectomy was obtained.

## Intervention

**FIG. 2.**
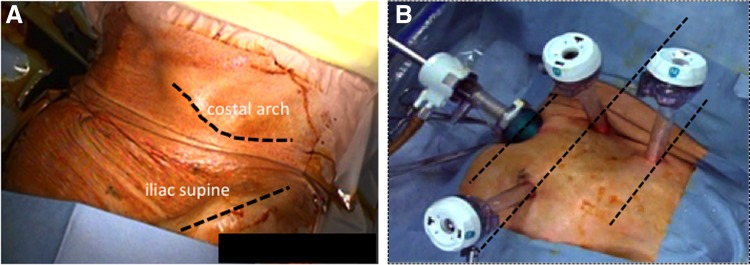
**(A)** The patient in the semilateral position with the back on the cushion. **(B)** Port placement.

**FIG. 3.**
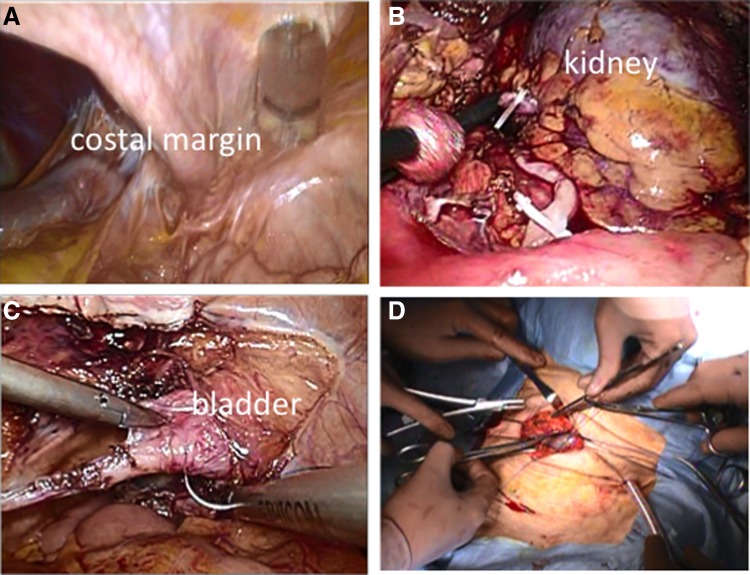
**(A)** Laparoscopic view. Working space was divided by left costal arch. Small intestine and descending colon were adhesive to the abdominal wall. **(B)** Nephrectomy was performed in a standard manner. **(C)** Suturing the edge of bladder before excision of bladder cuff. **(D)** The specimen was retrieved through the Pfannenstiel incision.

The patient was placed in the anteverted semilateral position with the right back on a cushion under general anesthesia (Fig. 2A). First, a 12-mm port was placed just under the umbilicus with the Hasson technique. The second and third 10-mm trocars were placed at 5 cm under the umbilicus and 3 cm above on the anterior axillary line. The fourth 10-mm trocar was at the umbilicus level, high on the middle axillary line (Fig. 2B). After the carbon dioxide pneumoperitoneum at 10 mm Hg pressure was induced, a small abdominal cavity and intestinal adhesion to the abdominal wall were observed. The first procedure was started with adhesiolysis of the small intestine and the descending colon. The colon was mobilized after incising along the white line of Toldt. There was the costal margin, which divided the working space and sometimes interrupted the laparoscopic procedures (Fig. 3A).

Procedures were mostly carried out using the medial to the lateral approach to avoid the costal margin. The anterior side of left kidney was exposed, and the renal vein and the artery were dissected properly. The ureter was dissected and clamped using hem-o-lok clips (Teleflex Co., Carlisle, SC) after the renal artery was clamped and cut (Fig. 3B). Complete nephrectomy was then performed in a standard manner. Dissection of the lower ureter and excision of bladder cuff were made by the laparoscopic procedure. The bladder was closed with intracorporeal single-layer continuous suturing using a barbed V-Loc™ 3-0 suture (Medtronic Co., Minneapolis, MN) (Fig. 3C). Integrity of the bladder closure was checked with saline instillation through a Foley catheter. The specimen was placed in the pouch and retrieved through a 5 cm Pfannenstiel incision (Fig. 3D). A drain was indwelling through the lower 10 mm port and the wound of remaining port was closed.

**FIG. 4.**
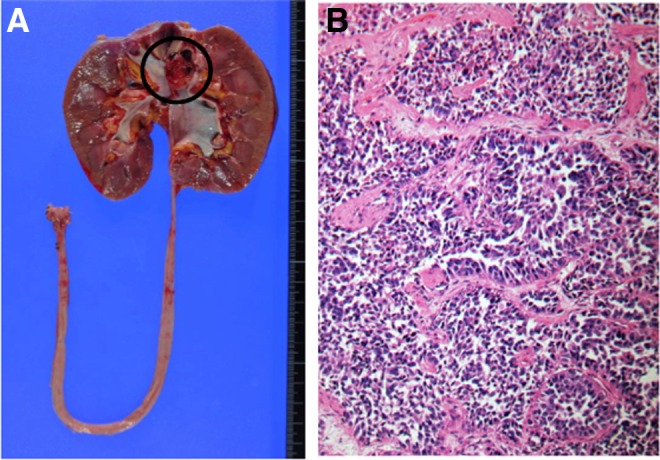
**(A)** The resected specimen. *Black* label demonstrates the lesion in renal pelvis. **(B)** Histopathologic image showed high-grade urothelial carcinoma.

The total operative time was 262 minutes with a blood loss of 141 mL. The patient was discharged on the seventh postoperative day without any complications. The pathology report revealed that the tumor was high-grade urothelial carcinoma, stage pT3, and the respective margin was negative (Fig. 4). The patient has rejected adjuvant chemotherapy consisting of gemcitabine and cisplatin, however, no evidence of recurrence was shown within a year by cystoscopy and CT scan.

## Discussion

The kyphosis is usually described as the deformity of the spine that results in an abnormally round back and can occur at any age due to osteoporosis, tuberculous spondylitis, congenital disease, infection related, tumor related, and other reasons.^1^ The thoracic cage is often tilted anteriorly, resulting in low-lying costal margins and narrow abdomen. Patients with kyphosis form a challenging group to laparoscopic surgeons because of difficulties in positioning and abdominal approach. The narrow abdomen causes difficulty with trocar insertion and some operative procedures. In addition, the low-lying costal margin can divide the operative field.

A literature search of PubMed database between 1990 and 2015 retrieved seven cases of laparoscopic surgery performed in patients with severe kyphosis, all had low-lying costal margins.^2,3^ The kinds of laparoscopic surgeries were three cholecystectomies, three hemicolectomies, and one sigmoidectomy.^2^ In all cases excepting one, the low-lying costal margin divided the operative field, resulting in a narrow working space after pneumoperitoneum was induced. The narrow abdomen can also cause difficulty in trocar insertion and in some operative procedures. Therefore, laparoscopic surgery with reduced port has been reported in dealing with a small working place in the abdomen.^3^ Surgical meticulous considerations in these patients must be taken for the abnormalities of the vascular anatomy affected by spinal curve.^3,4^ Kyphotic patients may also suffer considerable perioperative morbidity such as cardiac problems and respiratory insufficiency.^4^

To our knowledge, this is the first clinical case report of transperitoneal complete laparoscopic nephrouretectomy in a case of kyphosis. In our case, port placement was made at the lower abdominal wall than usual because the chest of the patient was tilted anteriorly and abdominal cavity was narrowed by low-lying costal margin. However, enough working space was obtained after pneumoperitoneum to perform nephroureterectomy, even though adhesion of the intestine to the abdominal wall was more severe than can be expected. Moreover, the bladder was located just beneath the sigmoid colon, therefore, the situation made it easier to excise the bladder cuff.

Kyphosis is described as a low-lying costal arch, resulting in less working space and disturbance of surgical view through the laparoscope. However, the artifice of position and port placement makes it possible to perform laparoscopic nephroureterectomy for the patient with kyphosis.

## Abbreviation Used

CT: computed tomography
